# Sub-analysis of perfusion balloon predilatation with intracoronary nicorandil vs. distal protection for acute coronary syndrome: A comparative evaluation

**DOI:** 10.20407/fmj.2025-009

**Published:** 2025-11-05

**Authors:** Masataka Yoshinaga, Takashi Muramatsu, Takashi Uwatoko, Akane Miyazaki, Takuo Toriya, Yuji Matsuwaki, Masato Ishikawa, Yuko Ukai, Yohei Kobayashi, Katsuyoshi Ito, Hideaki Ota, Yoshihiro Sobue, Eiichi Watanabe, Hideo Izawa

**Affiliations:** 1 Department of Cardiology, Fujita Health University Bantane Hospital, Nagoya, Aichi, Japan; 2 Department of Cardiology, Cardiovascular Center, Fujita Health University Hospital, Toyoake, Aichi, Japan; 3 Faculty of Nursing, Fujita Health University Hospital, Toyoake, Aichi, Japan; 4 Department of Radiology, Fujita Health University Hospital, Toyoake, Aichi, Japan

**Keywords:** Percutaneous coronary intervention, Acute coronary syndrome, Complication, Slow-flow, Perfusion balloon

## Abstract

**Objectives:**

Slow-flow or no-reflow events occur as a complication during percutaneous coronary intervention for acute coronary syndrome (ACS). A previous study demonstrated that prolonged perfusion balloon (PB) predilatation combined with intracoronary administration of nicorandil attenuates this phenomenon. This subanalysis compared the efficacy of the PB approach with that of conventional distal protection (DP).

**Methods:**

The study had a retrospective, single-center, observational design and included patients who underwent percutaneous coronary intervention for ACS between April 2020 and April 2022. The patients were divided into a PB group and a DP group. The PB group underwent thrombus aspiration, followed by 3 minutes of predilatation using the Ryusei^®^ PB, with simultaneous intracoronary administration of nicorandil 2 mg without a DP device and subsequent placement of a drug-eluting stent. The DP group underwent direct stenting or predilatation with a standard balloon combined with a DP device without a PB. Primary endpoints included the incidence of slow-flow or no-reflow events.

**Results:**

Sixty-four patients with ACS were enrolled (PB group, n=32; DP group, n=32). The incidence of slow-flow or no-reflow events was significantly lower in the PB group (3.1% vs. 46.8%, p<0.01), as was procedure time (87.1±26.4 minutes vs. 108.6±42.3 minutes, p=0.02) and volume of contrast used (186.0±65.0 mL vs. 228±75.8 mL, p=0.02).

**Conclusions:**

PB predilatation with intracoronary nicorandil significantly reduced the number of slow-flow or no-reflow events, shortened procedure time, and reduced the volume of contrast used. This method could be at least as effective as DP.

## Introduction

**Figure 1:**
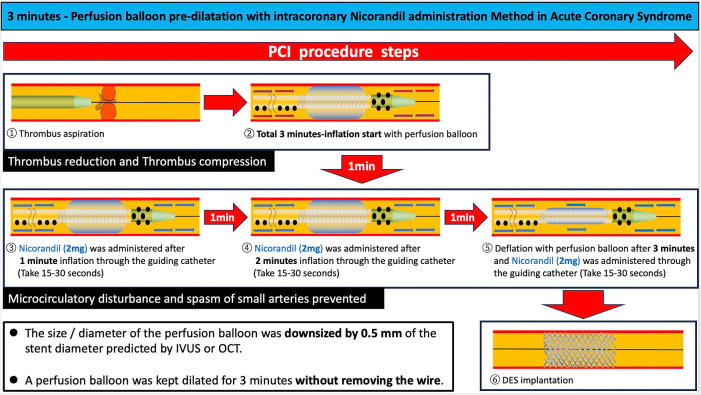
3 minutes – Perfusion balloon pre – dilatation with intracoronary nicorandil administration We perform thrombus aspiration to remove any floating aspirable thrombus, followed by inflating the perfusion balloon for 3 minutes to compress the thrombus and plaque against the vessel wall. During balloon inflation, vasodilators flow distally, so we hypothesize that injecting nicorandil into the coronary artery during balloon inflation can prevent small artery spasms and maintain microcirculation throughout the procedure, benefiting ACS patients by reducing the risk of slow flow or no-reflow.

Percutaneous coronary intervention (PCI) is the standard treatment for acute coronary syndrome (ACS). However, slow flow or no reflow during the procedure remains a significant complication.^1,2^ This phenomenon is caused primarily by microvascular dysfunction, microvascular obstruction, distal embolization, and vasospasm, and can substantially affect both short-term and long-term clinical outcomes.^3^ While slow-flow or no-reflow events are associated with increased mortality, no definitive solution has been established.^4,5^ One method for preventing slow flow or no reflow is the use of a distal protection filter device. However, the landmark EMERALD trial did not demonstrate any clinical benefits from using this method, such as reduced infarct size or improved outcomes.^6^ The VAMPIRE 3 trial showed that in patients with ACS and high-risk plaque morphology, the use of a distal protection filter device significantly lowered the incidence of no reflow (the primary endpoint) and the risk of subsequent cardiovascular events when compared with conventional PCI.^7^ Nonetheless, it has been reported that a distal protection device is used in only about 5.3% of patients who undergo PCI for ACS in Japan.^8^ One reason for this low usage rate may be that some operators deem the device unnecessary based on imaging findings. Another contributing factor could be the complexity of using these devices, particularly for distal lesions. With these issues in mind, we hypothesized that a strategy combining a 3-minute predilatation period using a perfusion balloon and intracoronary administration of nicorandil might be beneficial for reducing the risk of slow-flow or no-reflow events during PCI.^9^ This approach has been reported to maintain coronary blood flow, mitigate endothelial and microvascular spasm, and promote stabilization of thrombus (Figure 1). Nicorandil is key in this regard, given its dual action as an ATP-sensitive potassium channel opener and a nitrate, conferring vasodilatory effects and decreasing microvascular resistance.^10^ Previous studies have suggested that this strategy may reduce the incidence of slow flow or no reflow.^9^ The present study builds on previous findings by providing additional insights into the role of predilatation using a perfusion balloon. The objective of this research was to determine whether the efficacy of a combination of inflation using a perfusion balloon and nicorandil is comparable to or superior to that of a distal protection filter device in terms of preventing slow-flow or no-reflow events, potentially simplifying the procedural approach for intervention in patients with ACS.

## Methods

### Study participants

**Figure 2:**
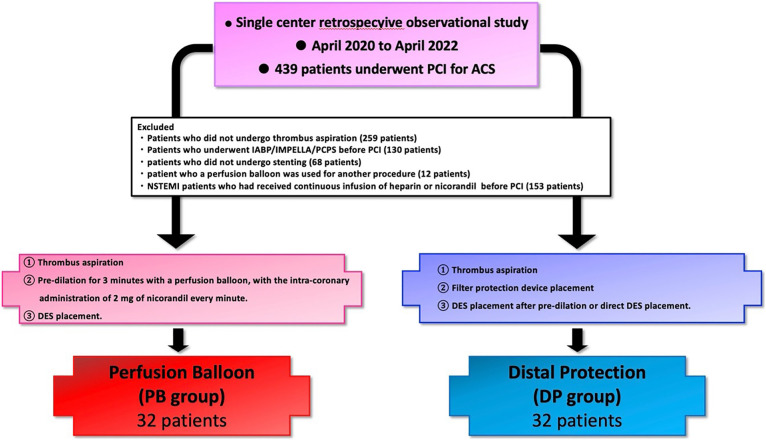
Study flow diagram

This retrospective, single-center observational study analyzed data from patients who presented with ACS (ST-elevation or non-ST-elevation myocardial infarction) and underwent urgent or emergent PCI at Fujita Health University Hospital between April 2020 and April 2022 (Figure 2). The dataset analyzed is the same as that in a previously published primary study entitled “A pilot study of perfusion balloon predilatation in conjunction with intracoronary nicorandil administration for acute coronary syndrome”.^9^ Baseline data were collected from electronic medical records and catheterization reports. The patients were divided into a perfusion balloon (PB) group (n=32) and a distal protection (DP) group (n=32). The following exclusion criteria were applied: no manual aspiration thrombectomy; no stent implantation; pre-procedure administration of intravenous nicorandil or heparin infusion for other indications; need for a mechanical circulatory support device (e.g., an intra-aortic balloon pump [IABP], percutaneous left ventricular assist device [Impella^®^], or veno-arterial extracorporeal membrane oxygenation) before the procedure (Figure 2). The need for written informed consent for inclusion in this study was waived because of the retrospective nature of the research. This subanalysis represents part of an ongoing evaluation of various techniques for preventing slow-flow or no-reflow events.

### Procedural details

The PB group was treated with predilatation using only a perfusion balloon without a distal protection device followed by implantation of a drug-eluting stent (DES). In the PB group, after aspiration of the thrombus, the size (i.e., nominal diameter) of the perfusion balloon was downsized by 0.5 mm of the angiographic reference diameter or expected stent diameter. A perfusion balloon was kept inflated at the nominal pressure (6 atm) for 3 minutes without removing the wire. During inflation with the perfusion balloon, nicorandil (2 mg) was administered three times through the guiding catheter at one-minute intervals (total dose, 6 mg). This approach aimed to compress the thrombotic material, stabilize plaque, and maintain distal blood flow by continuous vasodilation (Figure 1). The DP group was treated by direct stenting or predilatation using a standard balloon, followed by implantation of a DES in a standardized manner.^8^ After passage of a guidewire through the culprit lesion, manual aspiration thrombectomy was performed for all patients included in the present study.

### Study endpoints

The primary endpoint in this subanalysis was the incidence of coronary flow disturbance. Coronary blood flow was graded according to the Thrombolysis In Myocardial Infarction (TIMI) criteria as follows: grade 0 (no perfusion), grade 1 (penetration without perfusion), grade 2 (partial perfusion), or grade 3 (complete perfusion). Slow flow was defined as TIMI flow grade 1 or 2 and no reflow as TIMI flow grade 0. The primary objective of this subanalysis was to compare the incidence of slow-flow or no-reflow phenomena between the PB group and the DP group. Secondary endpoints included procedure time, volume of contrast medium used, length of hospitalization, and clinical outcomes at 30 days and 12 months. These definitions excluded angiographic evidence of severe coronary artery dissection or vasospasm.^11^ The lowest TIMI flow grade throughout the procedure was recorded.

### Statistical analysis

Continuous variables are presented as mean±standard deviation and were compared between the two groups using the Student’s *t*-test or Mann–Whitney *U* test based on normality. Categorical variables are expressed as the percentage and were compared between groups using the chi-squared or Fisher’s exact test. Analyses were performed using commercially available JMP software (SAS Institute Inc., Cary, NC, USA). A p-value of <0.05 was considered statistically significant.

## Results

**Figure 3:**
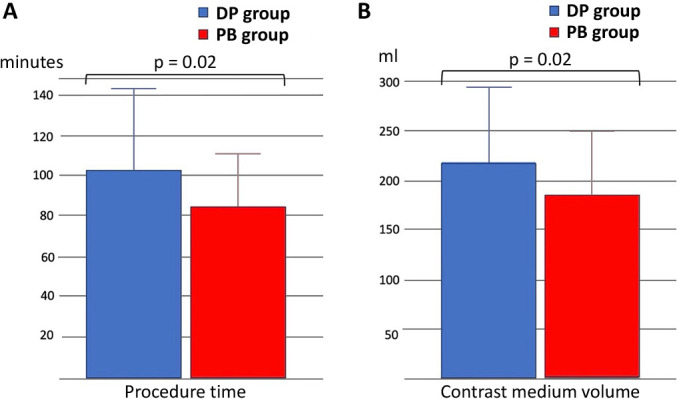
The procedure time (A) and the contrast medium volume (B)

**Figure 4:**
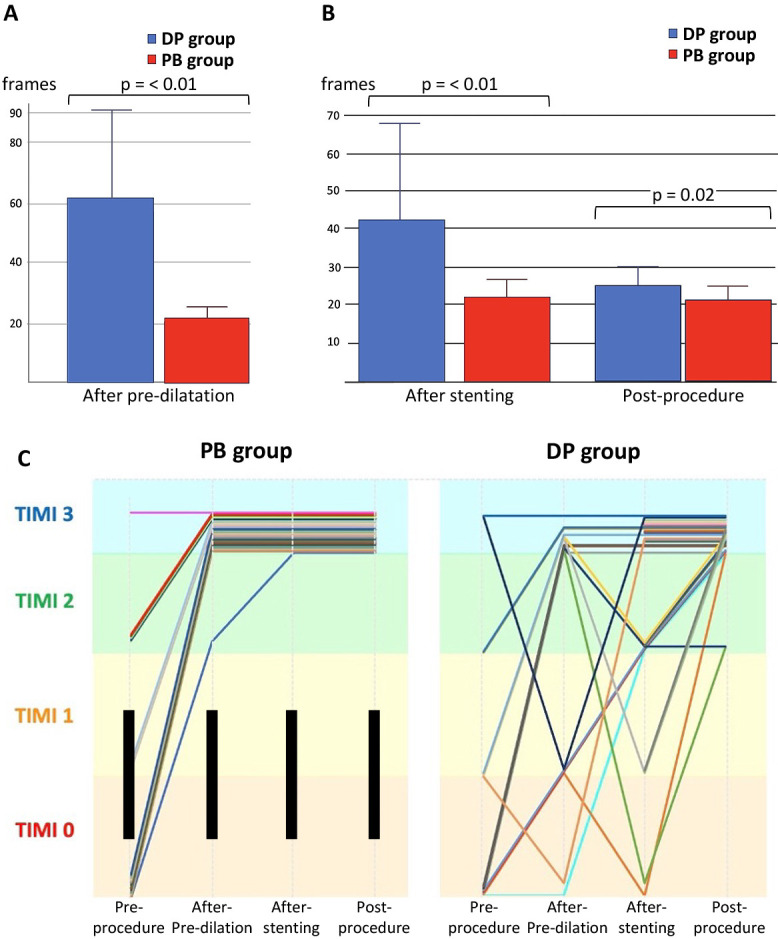
Corrected TIMI frame count (A) (B) and the rate of changes in the TIMI flow grade during the procedure (C)

**Figure 5:**
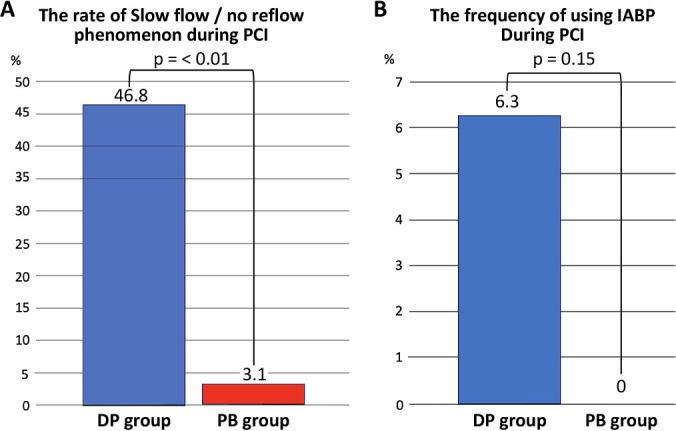
The rate of slow flow/ no reflow phenomenon (A) and the frequency of using IABP during PCI (B)

Data for 64 (14.5%) of the 439 patients who underwent emergent PCI for ACS during the study period were analyzed (PB group, n=32; DP group, n=32). The study flow diagram is shown in Figure 2. Baseline characteristics are shown in Table 1. The mean patient age was 70 years, and 81.2% of the participants were male. The average left ventricular ejection fraction before the procedure was 52.1% in the PB group and 50.0% in the DP group (p=0.27). There was no significant between-group difference in cardiac enzyme levels on admission or in the frequency of multivessel disease (40.6% vs. 34.4%, p=0.6). The majority of patients in both groups were classified as Killip class I (96.8% vs. 93.7%, p=0.35). Procedural characteristics are shown in Table 2. A 7 Fr guiding catheter via transradial access was the system most frequently used for primary PCI. Intracoronary imaging modalities were used in all cases; intravascular ultrasound was used in 71.8% and optical coherence tomography/optical frequency domain imaging in 28.2%. Manual aspiration thrombectomy was performed in all cases as per the exclusion criteria. In the DP group, 16 patients (50.0%) underwent direct stenting and 16 (50.0%) underwent predilatation with a balloon other than a perfusion balloon. The procedure time was significantly longer in the DP group than in the PB group (108.6±42.3 minutes vs. 87.1±26.4 minutes, p=0.02) (Figure 3A). The volume of contrast used was significantly greater in the DP group (228±75.8 mL vs. 186.0±65.0 mL, p=0.02) (Figure 3B). Figure 4 demonstrates the impact of perfusion balloon predilatation by showing a significant improvement in the corrected TIMI frame count after predilatation and stenting. The corrected TIMI frame count was significantly smaller in the PB group than in the DP group after predilatation (21.7±5.0 frames vs. 62.4±32.6 frames, p<0.01) and post-procedure (21.2±3.7 frames vs. 24.1±5.9 frames, p=0.02) (Figure 4A, 4B). One patient (3.1%) in the PB group temporarily experienced slow flow (i.e., TIMI flow grade 2). Blood flow was rapidly restored with vasodilators in this patient. In the DP group, 43.7% of the seven patients who underwent predilatation did not achieve TIMI flow grade 3. There was no significant difference in the final rate of achievement of TIMI grade 3 between the groups (100% vs. 93.7%) (Figure 4C). The incidence of slow flow or no reflow during the procedure was significantly higher in the DP group than in the PB group (46.8% vs. 3.1%, p<0.01) (Figure 5A). The significant reduction in slow-flow/no-reflow events suggests that perfusion balloon inflation with nicorandil has a meaningful clinical benefit, particularly in patients with high-risk ACS. Table 3 shows the results of the imaging analyses. There were no significant differences in geometric measurements (e.g., pre-procedural plaque area and post-procedural minimum stent area) or in the prevalence of plaque rupture, tissue protrusion after stenting, or major stent edge dissection between the two groups. IABP was not required during the procedure in any patient in the PB group but was needed in 6.3% of patients in the DP group (p=0.15) (Figure 5B). The clinical outcomes are shown in Table 4. There was no significant difference in length of hospitalization, peak creatine kinase level between the two groups. ST-segment resolution was significantly more often complete in the PB group than in the DP group (81.2% vs. 50.0%, p=0.03). No major adverse cardiac or cerebrovascular events were observed during hospitalization or at 30 days or 12 months in either group (Table 5).

## Discussion

Distal microthrombotic embolization, microvascular dysfunction, microvascular vasospasm, and reperfusion injury may play important roles in slow-flow or no-reflow events.^11^ The findings of this subanalysis underscore the efficacy of perfusion balloon predilatation with concomitant intracoronary administration of nicorandil in minimizing these events in patients with ACS. The slow-flow or no-reflow rate was markedly lower in the PB group (3.1%) than in the DP group (46.8%), aligning with the hypothesis that prolonged balloon inflation with stable blood flow and targeted pharmacologic vasodilation can reduce the risks of distal embolization and microvascular spasm. The higher incidence of intraprocedural slow-flow/no-reflow events observed in the DP group might be partially attributable to the presence of debris or thrombus trapped by the distal protection device itself. The absence of significant differences in final angiographic outcomes indicates that these devices effectively capture embolic debris, subsequently restoring optimal flow before final imaging. The mechanism explaining the reduced incidence of coronary flow disturbance in the PB group could be multifactorial. The following potential mechanisms can be considered.

### Continuous perfusion and thrombus stabilization

The Ryusei perfusion balloon allows for some degree of antegrade flow during inflation, which may reduce distal embolization of thrombus fragments. Prolonged inflation of a perfusion balloon might be more effective in terms of compressing and stabilizing the local atherothrombotic materials towards the vessel wall without compromising blood flow distal to the culprit lesion.

### Nicorandil-induced vasodilation

Nicorandil has a dual mechanism of action (nitrate-like attenuated platelet aggregation and neutrophil adhesion that promote capillary plugging and opening mitochondrial K^+^ATP-sensitive potassium channels) that likely augments coronary and microvascular vasodilation, improving microcirculatory perfusion and reducing the risk of ischemic injury.^10,12^ The hybrid pharmacological properties of nicorandil reduce microvascular resistance and increase coronary blood flow.^13^ A recent meta-analysis indicated that nicorandil significantly improves no flow and attenuates major adverse cardiac events in patients undergoing primary PCI.^14^

### Shorter procedure time and use of less contrast medium

With better initial preparation of the lesion, operators may achieve optimal stent expansion with less need for additional interventions, reducing the total procedure time and contrast load. While distal protection devices theoretically capture atherothrombotic debris, practical limitations (e.g., device crossing difficulties, additional steps) can increase the procedure time. The shorter procedural time and reduced contrast volume used in the PB group could be attributed to better initial preparation of the lesion through prolonged balloon inflation and the simultaneous vasodilation provided by nicorandil. This initial optimization may reduce the need for repeated balloon inflations or additional interventions, thereby shortening the procedure time and reducing the amount of contrast used. Manipulation of the device in the absence of distal protection steps is also likely to reduce the complexity of the procedure.

Given these findings, the perfusion balloon strategy with nicorandil may be considered a viable alternative to distal protection, particularly in cases where DP devices are challenging to deploy. We hypothesized that a 3-minute dilation with a perfusion balloon combined with intracoronary administration of nicorandil would reduce the risk of slow flow or no-reflow in patients with ACS when compared with direct stenting. Our previous study^9^ included 36 patients in the PB group and 51 in the direct stenting group. The incidence of slow flow or no reflow was significantly lower in the PB group than in the DS group (2.8% vs. 23.5%, p<0.01). An IABP was required in six patients (11.7%) in the DS group but not in any patient in the PB group (p<0.01).^9^ Nishino et al. compared the effectiveness of prolonged inflation of a Ryusei perfusion balloon (for 5 minutes) in conjunction with intracoronary administration of nitroprusside with that of a conventional PCI procedure before stenting in the setting of ST-elevation myocardial infarction.^15^ After propensity score matching, the incidence of slow-flow and no-reflow events was significantly lower, and post-procedural TIMI flow grade 3 was more frequently achieved in the PB group than in the conventional PCI group. These results were generally in line with those of the present study, suggesting that a perfusion balloon may have an advantage over a conventional balloon with respect to predilatation for better management of intracoronary thrombus and/or the vasoconstriction responsible for ACS. However, these studies did not evaluate whether use of a perfusion balloon is as effective as distal protection for prevention of distal embolization. The 20-mm length of the Ryusei perfusion balloon, which can cover attenuated plaque with a longitudinal length, may also be an effective factor. In this study, the incidence of slow-flow and no-reflow events was significantly lower in the PB group (3.1% vs. 46.8%, p<0.01), with a shorter procedure time (87.1±26.4 min vs. 108.6±42.3 min, p=0.02), and use of less contrast medium (186.0±65.0 mL vs. 228±75.8 mL, p=0.02). An IABP was needed in two patients (6.3%) in the DP group but not in any of those in the PB group (p=0.15). Moreover, clinical outcomes during hospitalization and at 3 and 12 months were similar between the PB and DP groups. Of note, the study population may have been highly selected, considering that patients with hemodynamic instability requiring mechanical circulatory support before PCI were excluded. The lower incidence rate of coronary flow impairment, the shorter procedure time, the use of less contrast medium, and the decreased need for mechanical circulatory support in the PB group suggest that this approach may not only help prevent hemodynamic deterioration during the procedure but also reduce the risk of post-procedural complications. An important point to note in this study is that no adverse events, such as atrioventricular block or ventricular tachycardia, were observed during intracoronary administration of nicorandil.

Our results suggest that this novel strategy of inflating a perfusion balloon for 3 minutes following aspiration of thrombus and injecting 2 mg of intracoronary nicorandil at 1-minute intervals may help to avoid the use of filter devices, complex procedures, and the risk of slow flow or no reflow. When treating ACS, this approach could potentially reduce the need for unnecessary mechanical circulatory support devices, minimize unnecessary radiation exposure, and decrease the amount of contrast agent used. Therefore, this novel approach could serve as a safe and effective alternative, offering comparable or even superior outcomes to distal protection in patients with ACS. However, further studies are required to clarify the clinical application of this strategy and to identify the subset of patients or lesions in which its use would be appropriate.

### Future perspectives

Additional multicenter randomized clinical trials should evaluate the perfusion balloon-plus-nicorandil approach across diverse ACS populations. Investigations into optimal balloon inflation time, balloon size, nicorandil dosing regimens, and the need for manual aspiration thrombectomy and adjunctive pharmacotherapy (e.g., antiplatelet or anticoagulant strategies) may further refine this technique. Moreover, advanced imaging modalities (such as optical coherence tomography or intravascular ultrasound) could offer deeper insights into the mechanistic benefits of prolonged balloon inflation with administration of nicorandil.

### Limitations

This study had several limitations. First, it had a retrospective single-center design and a relatively small sample size, which may limit the generalizability of its findings. Larger prospective studies are warranted. Second, device selection and procedural strategies depended on operator experience and preference, which could have introduced a degree of operator-dependent bias. Finally, the study participants were not randomized, and unmeasured confounders could have influenced outcomes.

## Conclusions

Perfusion balloon predilatation combined with intracoronary nicorandil markedly reduced the incidence of slow-flow and no-reflow events, shortened the procedure time, and decreased the volume of contrast used in comparison with interventions involving distal protection in ACS. This strategy may serve as a practical and efficacious alternative to distal protection. Further large-scale studies are needed to validate our findings and explore potential benefits in broader patient populations. This manuscript is based on a subanalysis incorporating data from Fujita Health University Hospital. All authors approved the final version of the manuscript.

## Figures and Tables

**Table 1:** Patient and lesion characteristics

Variable	PB group (n=32)	DP group (n=32)	p-value
Age, years	69.7±15.6	71.3±9.0	0.60
Male sex, n (%)	22 (68.8)	28 (87.5)	0.06
Body mass index, kg/m^2^	23.4±3.2	23.7±3.7	0.82
Hypertension, n (%)	23 (71.9)	26 (78.8)	0.51
Diabetes mellitus, n (%)	9 (28.1)	11 (33.3)	0.65
Dyslipidemia, n (%)	17 (53.1)	15 (45.5)	0.53
Current smoker, n (%)	18 (56.3)	9 (28.1)	0.02
Family history of CAD, n (%)	2 (6.3)	1 (3.1)	0.54
Prior myocardial infarction, n (%)	1 (3.1)	2 (6.3)	0.57
Prior bypass graft surgery, n (%)	0 (0.0)	0 (0.0)	N/A
Atrial fibrillation, n (%)	0 (0.0)	3 (9.3)	0.07
Systolic blood pressure, mmHg	132.0±42.0	154.0±36.3	0.33
Diastolic blood pressure, mmHg	66.0±22.9	81.2±18.9	0.20
LVEF, %	52.1±1.4	50.0±1.3	0.27
Hemoglobin, g/dL	13.9±1.9	14.7±1.8	0.07
eGFR, mL/min/1.73 m^2^	68.0±20.1	57.0±23.9	0.05
HbA_1c_, %	6.5±1.0	6.5±1.4	0.95
Triglycerides, mg/dL	73.5±80.8	62.2±59.4	0.53
HDL-C, mg/dL	37.5±11.0	36.2±8.8	0.60
LDL-C, mg/dL	94.1±33.4	107.0±31.1	0.11
CK, U/L	390±582	383±521	0.96
CK-MB, ng/dL	34.1±66.0	42.4±63.6	0.60
Troponin I, pg/mL	4.8±10.7	9.9±33.0	0.42
Clinical presentation, n (%)			0.39
ST-segment elevation MI	28 (87.5)	30 (93.7)	
Non-ST segment elevation MI	4 (12.5)	2 (6.3)	
Unstable angina	0 (0.0)	0 (0.0)	
Killip class on admission, n (%)			0.35
I	31 (96.8)	30 (93.7)	
II	1 (3.1)	2 (6.2)	
III	0 (0.0)	0 (0.0)	
IV	0 (0.0)	0 (0.0)	
Culprit vessel, n (%)			0.43
Right coronary artery	16 (50.0)	11 (34.3)	
Left anterior descending	14 (43.8)	19 (59.4)	
Left circumflex	2 (6.3)	2 (6.3)	
In-stent restenosis, n (%)	0 (0.0)	0 (0.0)	N/A
Multivessel disease, n (%)	13 (40.6)	11 (34.4)	0.60
Medications on admission, n (%)			
Aspirin	3 (9.4)	6 (18.2)	0.30
Clopidogrel	0 (0.0)	0 (0.0)	N/A
Prasugrel	0 (0.0)	0 (0.0)	N/A
Oral anticoagulants	0 (0.0)	1 (3.0)	0.29
ACE inhibitors, ARB, or ARNI	11 (34.4)	16 (50.0)	0.25
β-blockers	4 (12.5)	6 (18.2)	0.53
Calcium channel blockers	10 (31.3)	13 (39.4)	0.49
Statins	10 (31.3)	8 (25.0)	0.40
Ezetimibe	3 (9.4)	0 (0.0)	0.07
Nitrates	1 (3.1)	2 (6.0)	0.57
Nicorandil	0 (0.0)	0 (0.0)	N/A
Medications at discharge, n (%)			
Aspirin	32 (100)	32 (100)	1.00
Clopidogrel	1 (3.1)	1 (3.1)	1.00
Prasugrel	31 (96.8)	31 (96.8)	1.00
Oral anticoagulants	0 (0.0)	3 (9.3)	0.07
ACE inhibitors, ARB, or ARNI	24 (75.0)	23 (71.8)	0.77
β-blockers	28 (87.5)	29 (90.6)	0.68
Calcium channel blockers	4 (12.5)	7 (21.8)	0.32
Statins	32 (100)	32 (100)	1.00
Ezetimibe	20 (62.5)	22 (68.7)	0.59
Nitrates	7 (21.8)	7 (21.8)	1.00
Nicorandil	12 (33.3)	10 (19.6)	0.14

The data are shown as the mean standard deviation or number (percentage) as appropriate. ACE, angiotensin-converting enzyme; ARB, angiotensin receptor blocker; ARNI, angiotensin receptor neprilysin inhibitor; CAD, coronary artery disease; CK, creatine kinase; CK-MB, creatine kinase myocardial band; DP, distal protection; eGFR, estimated glomerular filtration rate; HbA_1c_, glycated hemoglobin; HDL-C, high-density lipoprotein cholesterol; LDL-C, low-density lipoprotein cholesterol; LVEF, left ventricular ejection fraction; MI, myocardial infarction; PB, perfusion balloon

**Table 2:** Procedural details

Variables	PB group (n=32)	DP group (n=32)	p-value
Vascular access, n (%)			0.20
Radial	28 (87.4)	27 (84.4)	
Brachial	2 (6.3)	0 (0.0)	
Femoral	2 (6.3)	5 (15.6)	
Size of guiding catheter, n (%)			1.00
6 Fr	2 (6.3)	2 (6.3)	
7 Fr	30 (93.7)	30 (93.7)	
8 Fr	0 (0.0)	0 (0.0)	
Intracoronary imaging modality, n (%)			
IVUS	25 (78.1)	21 (65.6)	0.27
OCT or OFDI	7 (21.9)	11 (34.4)	0.27
Manual aspiration thrombectomy, n (%)	32 (100)	32 (100)	1.00
Distal filter protection device, n (%)			
Filtrap	0 (0)	3 (9.4)	N/A
Parachute	0 (0)	29 (90.6)	N/A
Direct stent, n (%)	0 (0)	16 (50.0)	<0.01
Predilatation, n (%)	32 (100)	16 (50.0)	<0.01
Predilatation balloon, n (%)	(n=32)	(n=16)	<0.01
Conventional	0 (0.0)	12 (75%)	
Modified	0 (0.0)	4 (25%)	
Perfusion	32 (100)	0 (0.0)	
Predilatation balloon size, mm	(n=32)	(n=16)	
	2.9±0.5	2.5±0.6	<0.01
Predilatation balloon inflation pressure, atm	(n=32)	(n=16)	
	7.4±2.1	9.5±3.6	0.02
Predilatation balloon inflation time, seconds	(n=32)	(n=16)	
	180±0.0	32.4±19.6	<0.01
Drug-eluting stent, n (%)			0.08
XIENCE	4 (12.5)	8 (25.0)	
SYNERGY	8 (25.0)	2 (6.3)	
Resolute Onyx	10 (31.3)	8 (25.0)	
Ultimaster	5 (15.6)	2 (6.3)	
Orsiro	4 (12.5)	11 (34.4)	
Combo	1 (3.1)	1 (3.1)	
Stent diameter, mm	3.4±0.5	3.4±0.5	0.90
Stent length, mm	28.5±9.2	30.0±9.3	0.50
Maximum stent inflation pressure, atm	10.8±1.5	11.6±2.9	0.14
Corrected TIMI frame count, frames	(n=32)	(n=16)	
After predilatation	21.7±5.0	62.4±32.6	<0.01
Corrected TIMI frame count, frames			
After stenting	22.0±4.4	42.5±25.5	<0.01
Post-procedure	21.2±3.7	24.1±5.9	0.02
TIMI flow grade pre-procedure			0.37
0	20 (62.5)	13 (40.6)	
1	5 (15.6)	7 (21.9)	
2	6 (18.8)	10 (31.2)	
3	1 (3.1)	2 (6.3)	
TIMI flow grade after predilatation	(n=32)	(n=16)	<0.01
0	0 (0.0)	2 (12.5)	
1	0 (0.0)	4 (25.0)	
2	1 (3.1)	1 (6.2)	
3	31 (96.9)	9 (56.3)	
TIMI flow grade after stent implantation			<0.01
0	0 (0.0)	2 (6.2)	
1	0 (0.0)	3 (9.4)	
2	0 (0.0)	8 (25.0)	
3	32 (100)	19 (59.4)	
TIMI flow grade post-procedure			0.15
0	0 (0.0)	0 (0.0)	
1	0 (0.0)	0 (0.0)	
2	0 (0.0)	2 (6.3)	
3	32 (100)	30 (93.7)	
Incidence of slow-flow or no-reflow	1 (3.1)	15 (46.8)	<0.01
Onset-to-door time, minutes	150±102	214±244	0.27
Door-to-balloon time, minutes	85±39	79±47	0.59
Procedure time, minutes	87.1±26.4	108.6±42.3	0.02
Total dose of nicorandil, mg	10.0±2.3	8.1±4.9	0.04
Total dose of isosorbide dinitrate, mg	2.3±0.7	3.5±1.9	<0.01
Total dose of nitroprusside, mg	0.0±0.0	1.8±5.3	0.07
Volume of contrast medium, mL	186.0±65.0	228±75.8	0.02

The data are shown as the mean standard deviation or number (percentage) as appropriate. DS, direct stenting; IVUS, intravascular ultrasound; OCT, optical coherence tomography; OFDI, optical frequency domain imaging; PB, perfusion balloon; TIMI, thrombolysis in myocardial infarction

**Table 3:** Findings on imaging

Variable	PB group	DP group	p-value
Quantitative coronary angiography	(n=32)	(n=32)	
Pre-procedure			
Reference vessel diameter, mm	3.0±0.4	3.0±0.6	0.91
Minimum lumen diameter, mm	0.5±0.6	0.6±0.6	0.62
Diameter stenosis, %	89.3±17.8	86.1±15.2	0.43
Lesion length, mm	25.7±9.8	22.8±11.8	0.29
Visible thrombus, n (%)	27 (84.3)	26 (81.2)	0.74
Post-procedure			
Reference vessel diameter, mm	2.9±0.5	3.1±0.6	0.21
Minimum lumen diameter, mm	2.4±0.5	2.2±0.5	0.13
Diameter stenosis, %	10.9±6.1	12.7±9.8	0.36
Visible thrombus, n (%)	0 (0.0)	1 (3.1)	0.31
IVUS	(n=25)	(n=21)	
Pre-procedure			
Vessel area, mm^2^	13.0±3.9	13.3±3.3	0.76
Plaque area, mm^2^	11.4±3.8	11.3±3.1	0.95
Minimum lumen area, mm^2^	1.5±0.5	1.7±0.4	0.07
Area of stenosis, %	86.5±5.9	85.6±5.3	0.56
Length of attenuated plaque, mm	12.7±3.9	13.6±5.2	0.51
Plaque rupture, n (%)	23 (92.0)	19 (90.4)	0.85
Post-procedure			
Minimum stent area, mm^2^	7.1±1.8	7.0±1.8	0.95
Tissue protrusion, n (%)	2 (8.0)	4 (19.0)	0.26
Major stent edge dissection, n (%)	0 (0.0)	0 (0.0)	1.00
OCT or OFDI	(N=7)	(N=11)	
Pre-procedure			
Minimum lumen area, mm^2^	1.2±0.4	1.0±0.3	0.32
Ruptured fibrous cap, n (%)	5 (71.4)	9 (81.8)	0.60
Length of attenuated plaque, mm	12.9±4.3	9.8±3.0	0.08
Intact fibrous cap, n (%)	2 (28.6)	2 (18.1)	0.60
Thrombus, n (%)	7 (100)	11 (100)	1.00
Post-procedure			
Minimum stent area, mm^2^	6.2±1.4	6.4±1.5	0.87
Tissue protrusion, n (%)	3 (42.9)	6 (54.6)	0.62
- Smooth	3 (42.9)	5 (45.4)	0.91
- Disrupted fibrous	0 (0.0)	0 (0.0)	1.00
- Irregular	0 (0.0)	1 (9.09)	0.41
Major stent edge dissection, n (%)	0 (0.0)	0 (0.0)	1.00

The data are shown as the mean standard deviation or number (percentage) as appropriate. DP, distal protection; IVUS, intravascular ultrasound; OCT, optical coherence tomography; OFDI, optical frequency domain imaging; PB, perfusion balloon

**Table 4:** Clinical outcome measures

Variables	PB group (n=32)	DP group (n=32)	p-value
Length of hospitalization, days	11.6±4.3	14.4±7.7	0.08
Peak CK level, U/L	1861±1862	1960±1766	0.83
Pre-procedure Σ STE, mm	6.2±2.9	6.6±3.0	0.56
Post-procedure Σ STE, mm	1.5±1.1	2.5±1.8	0.08
ΔChange in Σ STE, mm	4.7±2.8	4.0±2.7	0.17
ST-segment resolution, n (%)			0.03
Complete	26 (81.2)	16 (50.0)	
Partial	3 (9.4)	9 (28.1)	
None	3 (9.4)	7 (21.9)	
Mechanical circulatory support, n (%)			
IABP	0 (0.0)	2 (6.3)	0.15
Impella	0 (0.0)	0 (0.0)	N/A
VA-ECMO	0 (0.0)	0 (0.0)	N/A
In-hospital outcomes, n (%)			
Cardiac death	0 (0.0)	0 (0.0)	N/A
Non-cardiac death	0 (0.0)	0 (0.0)	N/A
MI in target vessel	0 (0.0)	0 (0.0)	N/A
Revascularization of target vessel	0 (0.0)	0 (0.0)	N/A
Stent thrombosis	0 (0.0)	0 (0.0)	N/A
Disabling ischemic stroke	0 (0.0)	0 (0.0)	N/A

The data are shown as the mean standard deviation or number (percentage) as appropriate. CK, creatine kinase; DP, distal protection; IABP, intra-aortic balloon pump; MI, myocardial infarction; PB, perfusion balloon; STE, ST-segment elevation; VA-ECMO, veno-arterial extracorporeal membrane oxygenation

**Table 5:** Clinical outcomes at 30 days and 12 months

Variables	PB group (n=32)	DP group (n=32)	p-value
Cardiac deaths at 30 days, n (%)	0 (0.0)	0 (0.0)	N/A
Cardiac deaths at 12 months, n (%)	0 (0.0)	0 (0.0)	N/A
Non-cardiac deaths at 30 days, n (%)	0 (0.0)	0 (0.0)	N/A
Non-cardiac deaths at 12 months, n (%)	2 (6.2)	1 (3.1)	0.35
New-onset sustained hypotension within 30 days, n (%)	0 (0.0)	0 (0.0)	N/A
New-onset sustained hypotension within 12 months, n (%)	0 (0.0)	0 (0.0)	N/A
New-onset severe heart failure within 30 days, n (%)	1 (3.1)	3 (9.3)	0.30
New-onset severe heart failure within 12 months, n (%)	0 (0.0)	2 (6.2)	0.15
Readmission for left ventricular failure within 30 days, n (%)	1 (3.1)	2 (6.2)	0.55
Readmission for left ventricular failure within 12 months, n (%)	0 (0.0)	3 (9.3)	0.07
Reinfarction within 30 days, n (%)	0 (0.0)	0 (0.0)	N/A
Reinfarction within 12 months, n (%)	0 (0.0)	0 (0.0)	N/A
Ischemic target vessel revascularization within 30 days, n (%)	0 (0.0)	0 (0.0)	N/A
Ischemic target vessel revascularization within 12 months, n (%)	1 (3.1)	2 (6.2)	0.35
Disabling symptoms at 30 days, n (%)	0 (0.0)	0 (0.0)	N/A
Disabling symptoms at 12 months, n (%)	0 (0.0)	0 (0.0)	N/A
Composite MACE related to left ventricular dysfunction at 30 days, n (%)	0 (0.0)	2 (6.3)	0.15
Composite MACE related to left ventricular dysfunction at 12 months, n (%)	0 (0.0)	1 (3.1)	0.31

The data are shown as the mean standard deviation or number (percentage) as appropriate. DP, distal protection; MACE, major adverse cardiac events; PB, perfusion balloon

## Footnotes

The authors attest that they are in compliance with human studies committees, animal welfare regulations of the authors’ institutions, and Food and Drug Administration guidelines, including patient consent where appropriate.
